# Conceptualizations of clinical decision-making: a scoping review in geriatric emergency medicine

**DOI:** 10.1186/s12873-020-00367-2

**Published:** 2020-09-14

**Authors:** Maria Louise Gamborg, Mimi Mehlsen, Charlotte Paltved, Gitte Tramm, Peter Musaeus

**Affiliations:** 1 Centre for Health Sciences Education, Faculty of Health, Aarhus University, Aarhus, Denmark; 2 Corporate HR MidtSim, Central Region of Denmark & Department of Clinical Medicine, Faculty of Health, Aarhus University, Aarhus, Denmark; 3 Department of Psychology, Faculty of Business and Social Sciences, Aarhus University, Aarhus, Denmark

**Keywords:** Decision making, Geriatric patients, Clinical judgement, Scoping review, Biases and heuristics, Cognition, Young physicians

## Abstract

**Background:**

Clinical decision-making (CDM) is an important competency for young doctors especially under complex and uncertain conditions in geriatric emergency medicine (GEM). However, research in this field is characterized by vague conceptualizations of CDM. To evolve and evaluate evidence-based knowledge of CDM, it is important to identify different definitions and their operationalizations in studies on GEM.

**Objective:**

A scoping review of empirical articles was conducted to provide an overview of the documented evidence of findings and conceptualizations of CDM in GEM.

**Methods:**

A detailed search for empirical studies focusing on CDM in a GEM setting was conducted in PubMed, ProQuest, Scopus, EMBASE and Web of Science. In total, 52 publications were included in the analysis, utilizing a data extraction sheet, following the PRISMA guidelines. Reported outcomes were summarized.

**Results:**

Four themes of operationalization of CDM emerged: CDM as dispositional decisions, CDM as cognition, CDM as a model, and CDM as clinical judgement. Study results and conclusions naturally differed according to how CDM was conceptualized. Thus, frailty-heuristics lead to biases in treatment of geriatric patients and the complexity of this patient group was seen as a challenge for young physicians engaging in CDM.

**Conclusions:**

This scoping review summarizes how different studies in GEM use the term CDM. It provides an analysis of findings in GEM and call for more stringent definitions of CDM in future research, so that it might lead to better clinical practice.

## Background

Clinical Decision-Making (CDM) is an important part of medical education. Given young doctors’ limited experience, their CDM is more likely to be influenced by uncertainty [1, 2] and associated with errors [3]. However, a variety of definitions and operationalizations are seen across medical domains [1, 4],

Literature on CDM takes its point of departure from a variety of perspectives and approaches [5, 6], but CDM is commonly described as the formulation of hypotheses, diagnoses, and management plans in a systematic and structured process [4, 7–9]. Heuristics and biases [10], contextual factors [11], and bias-reduction [12] are emphasized in the literature. Taking a cognitive approach to understanding the processes underlying decisions [3, 10, 13], it focuses on the impact of decision-aids [14, 15], and medical errors [12, 16]. However, this cognitive approach to CDM arguably still struggles to link specific biases and errors. A review by Saposnik et al. [10] of this cognitively focused literature call for more empirical research into what contextual and social aspects moderates and mediates CDM [10]**.**

It is a challenge to investigate how and when clinical decisions are made [9, 17]. Clinical settings such as emergency departments (ED’s) that challenge physicians’ CDM may be the most optimal settings for investigations [18–21]. Furthermore, the decision-making literature underline how complex decisions are the most proficient at displaying the intricate structure of CDM [22]. Care for geriatric patients is complex [23, 24], as they are more prone to adverse outcomes [25], compared to other adult patients [26–29]. Geriatric-specific medical problems, e.g. multimorbidity [30] and biases, e.g. ageism [31], greatly impact CDM.

Existing reviews have focused on the characteristics of geriatric patients in the ED [32], the distribution of a priori decisions when consulting geriatric patients in the ED [24], or the impact of an assessment of geriatric patient’s cognitive abilities on health outcomes [33]. However, none of these reviews discusses how the notion of CDM is conceptualized in this body of research. Moreover, existing reviews do not find consistent results, which could be a consequence of the general confusion about how to describe and investigate CDM.

This scoping review therefore analyze the conceptualizations of CDM in terms of how it is defined and operationalized in empirical articles in GEM. The aim was to create an overview of the conceptualizations of CDM employed in the current empirical research in this domain. Thereby, we wished to clarify the conceptualization of the psychological aspects of CDM.

## Methods

### Eligibility criteria

A systematic search of terms related to CDM and Emergency Medicine (EM) was performed on title or abstract in PubMed, ProQuest, Scopus, EMBASE and Web of Science on 13th of March 2020. Terms related to geriatrics was searched in entire manuscripts to allow inclusion of studies, which did not exclusively address geriatric patients (see Table 1). Inclusion criteria were: EM, CDM, elderly patients, peer reviewed, empirical articles published in English or Scandinavian languages. To exclude articles using the term “decision-making” in everyday language (e.g. sentences like: “this has consequences for decision-making”), CDM was defined as a delineated construct, which can be moderated or mediated by factors in the clinical setting or inherent psychological factors within the clinician.

**Table 1:** Literature search strategy

Search string	Where
“Clinical Decision-Making” OR “CDM” OR “Clinical Decision Making” OR “Clinical Problem Solving” OR “Clinical Problem-Solving” OR “Medical Decision Making” OR “Medical Decision-Making” OR “Medical Problem Solving” OR “Medical Problem-Solving” OR Diagnostic Reasoning* OR Clinical Reasoning* OR Medical Reasoning* OR Medical Judgement* OR Clinical Judgement* OR Diagnostic Judgement* OR Diagnostic error*	Title and/or Abstract
Geriatric* OR Gero* OR Older Patient* OR Older adult* OR Elder* OR Geronto* OR Aged OR Aging OR Ageing OR Senior*	Anywhere
Emergency Medicine* OR Emergency Department* OR Emergency Ward* OR Emergency Team* OR Emergency Medical Team* OR Acute Medicine* OR “"Acute Medical Teams”" OR Acute Department* OR Acute Ward	Title and/or Abstract

Exclusion criteria were: (1) patient groups with a mean age ≤ 65 years, (2) non-clinician decision-making, (3) survey of opinions, (4) single case reports, (5) treatment evaluations (e.g. comparing risks, etc.), and (6) diagnostic errors not investigated in relation to CDM (e.g. type of errors associated with re-admission).

### Data collection process

Two blinded reviewers (MLG and GT) independently reviewed all studies in a standardized manner from agreed-upon exclusion guidelines. First, title and abstract were screened following the inclusion and exclusion criteria. After the initial screening, the two reviewers met to discuss and resolve discrepancies by consensus and discussions with other review group members (MM and PM). Second, the reviewers (MLG and GT) independently screened full text studies for eligibility, adhering to those same criteria. Three reviewers (MLG, PM and MM) then reviewed and discussed all included articles. Based on this initial review methods of analysis was agreed upon and a data extraction sheet inspired by the Cochrane Consumers and Communication Review Group’s data extraction template was then introduced. The first author (MLG) independently used this for charting, analysing, and synthesizing data from all included studies. Three reviewers (MLG, PM and MM) collaborated on the subsequent dataanalysis.

## Results

**Fig. 1:**
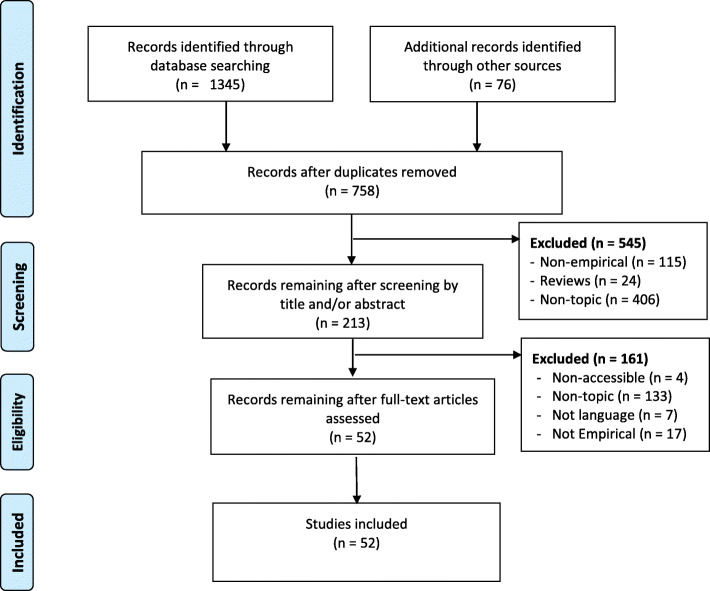
Study selection and PRISMA flowchart

Search criteria yielded 1421 publications. In total, 758 remained after duplicates were removed, from which 52 were included in the final analysis (see Fig. 1). Using PRISMA guidelines [34, 35], data was extracted from all 52 records. We identified study designs, type of settings, type of health care professionals participating in the study, and the age of the patients included. We then identified and grouped studies into themes of operationalizations of CDM and synthesized types of study objectives for each study within each operationalization. Lastly, we identified if and how studies defined CDM and grouped these into themes.

### Study characteristics

Overall study characteristics are presented in Additional file 1.

#### Designs

The design of the studies was distributed as follows: Quantitative study designs (*n* = 39), prospective observational studies (*n* = 21), retrospective observational studies (*n* = 6), intervention studies (*n* = 3), randomized controlled trials (*n* = 2), survey studies (*n* = 5), or experimental studies (*n* = 2).

Eleven publications described qualitative study designs; interview studies (n = 5), think-aloud studies (*n* = 2), case studies (n = 2) or ethnographic studies (*n* = 2).

The remaining two studies described mixed method study designs; a case study and value-stream mapping from an ethnographic observation study, or focus group interviews and an experimental study.

#### Settings

Most studies were conducted in EDs at teaching hospitals (*n* = 36). The remaining were conducted in urban, tertiary-care EDs (*n* = 14), in-patient ED wards (*n* = 3), or community hospitals (*n* = 6), or were performed outside a clinical setting, utilizing written ED cases (*n* = 3).

#### Health care professionals

The majority of studies (*n* = 42) investigated clinicians, from ED specialists to Post Graduate Year (PGY) 1 residents, including other medical specialties (neurologists and cardiologists) working in ED settings. Other health care staff, (nurses, dieticians, therapists, support workers, pharmacists and emergency medical technicians), were included in 14 studies. Five studies did not specify which health care professionals they included [36–40].

#### Geriatric patients

Generally, studies defined geriatric patients in terms of a mean age over 65 years, or otherwise stratified patients in age intervals, making it possible to extract results referring specifically to the geriatric group. However, some only mentioned elderly patients or presented case scenarios with geriatric patients, but did not specify age. These were included nonetheless.

### Four themes in operationalization of CDM

A large variation was found in how studies assessed CDM, possibly reflecting different conceptualizations. Therefore, it seemed necessary to record how studies operationalized CDM, in order to describe these different conceptualizations. We sorted the different operationalizations into subthemes, which we grouped in four overarching themes, presented with examples in Table 2 (see Additional file 2 for the full table).

**Table 2:** Coding of operationalizations of Clinical Decision-Making in geriatric emergency medicine

*Overarching theme*	*Subthemes*	*ID*	*Examples of operationalization of theme*
*CDM as dispositional decisions*	Observations of demonstrated binary CDM	19	“The decision to order physical restraint …” (P1280)
		42	“… followed by a question asking if the physician completing the questionnaire would cease or continue CPR under that set of circumstances.” (P12)
	Observations of demonstrated categorical CDM	6	Decision-making refer to which specific decision was made based on the clinical data available: “… there also were instances when the clinician decision making was contrary to the absence of an AMI.” (P1226)
*CDM as cognitive processes*	Cognitive: Illness scripts (networks of knowledge), Mental models, memory, judgement, human judgement/heuristic judgement/mental shortcuts, etc.	32	“Cognitive faculties deserve particular attention, as they are the bases of the clinical decision-making process … human abilities are limited and both gathering and retrieving information are inaccurate processes [2, 9]. Furthermore, in emergency medicine, “a priori” probabilities often are unknown, whereas missing data and ambiguities are frequent... This particular field favors intuitive and automatic tools as heuristics [1, 5].” (P2031)
		17	“Heuristics are mental shortcuts that often produce valid judgements but can lead to errors in atypical or rare events. Because they reflect natural processes, heuristics are not easily, or even productively, replaced.” (P9204)
	Knowledge and attitudes	3	“We designed a comprehensive written survey to assess ED provider knowledge, attitudes, and practice regarding placement of IUCs [including] team dynamics of decision making in UIC placement and management …” (P415)
		15	Refers to confidence, attitudes and knowledge, but does not address decision-making, specifically.
	Uncertainty	25	Diagnostic uncertainty: “… was quantified by a visual analogue scale (VAS) for ACS probability ranging from 0 to 100%.” (P29
*CDM as a model*	Statistical model/clinical decision rule	14	A decision-making analysis of certain risk stratification scores, as a statistical model.
	Decision rule and motivations/perception of utility	39	Validation of a decision rule and investigation of the motivations for certain decisions. They were surveyed about the latter.
		44	Describes decision-making only in terms of the decision-making support tool, but no other description.
*CDM as clinical judgement*	Clinical judgement: use of a structure/tool	43	“Upon final ED disposition, study staff administered a survey to the attending ED physician or senior resident querying the physician’s impression of the likelihood of an acute bacterial infection and the infections suspected on a 5-point Likert scale from very unlikely to very likely.” (P1803)
	Clinical Judgement: Practice as usual	37	“Because, to the best of our knowledge, no validated scoring system exists to quantify clinical judgement, we a priori chose to use the disposition decision of the treating physician in the ED as a proxy measure for clinical judgement …” (P294)
		24	“Clinical judgement can be defines as “an interpretation or conclusion about a patient’s needs, concerns, or health problems, and/or the decision to take action (or not), use or modify standard approaches, or improvise new ones as deemed appropriate by the patient’s response” [11]. It is complex and requires a flexible ability to recognise prominent aspects of an undefined clinical situation interpret their meaning and respond appropriately. It relates to the experience of individual clinicians.” (P5)

#### Theme 1: CDM as dispositional decisions (*n* = 11)

Within this theme, CDM was operationalized as the decision itself, by recording which decisions were made (i.e. the decision to cease CPR or not), and how specific decisions were influenced by provider characteristics (i.e. confidence, uncertainty, etc.), social or contextual factors.

#### Theme 2: CDM as cognition (*n* = 12)

The cognitive processes underlying CDM included ‘mental models’, thought processes, or mental processing, described as heuristics, perception, knowledge or attitudes. A common premise amongst these studies was that they *“...recognize [d] the salience of individual cognition, as well as [acknowledged] that the knowledge and experience that guides that cognition is constructed through social interaction and organizational context.”* [41] (p161).

#### Theme 3: CDM as a model (*n* = 7)

These studies primarily investigated how young physicians’ CDM was aided by rules, guidelines, or technologies, or how statistical models of risks improved predictability and aided decisions about diagnosis or treatment.

#### Theme 4: CDM as clinical judgement (*n* = 22)

The common term clinical judgement referred to *“ … the normal practice by [clinicians] using individual’s [clinical] knowledge, clinical expertise and gut feeling …*” *(p27)* [42]*.* Here, CDM was most often assessed through the clinician’s estimated probability of a certain clinical outcome or a final diagnosis. However, despite being a demarcated expression, CDM as Clinical Judgement was generally not defined in terms of a theoretical framework, with only one publication providing a description of the psychological behaviour of clinicians:

*“Clinical judgement … is complex and requires a flexible ability to recognise prominent aspects of an undefined clinical situation interpret their meaning and respond appropriately. It relates to the experience of individual clinicians.”* [43] *(p5).*

However, this description was not rooted in a theoretical framework.

### Relationship between operationalizations and study objectives

From this identification of operationalizations of CDM, it became relevant to link this to study objectives, in order to see if the different operationalizations organized meaningfully within specific aims of the research. By analyzing study aims in relation to CDM, we found that CDM was investigated in three study objectives:

1. Effects of **Aids (*n*** **= 33).**
2. Effects of **Cognitive processes or contextual factors (*n*** **= 14)**.
3. Effects of **Training or experience (*n*** **= 5)**.

By this comparison, we were able to provide a more comprehensive overview, demonstrating some tendencies amongst the empirical research in this field. This combined overview is presented in Table 3, representing each publication ID in both their theme of operationalization and type of study intervention.

**Table 3:** Study objectives organized within operationalizations of Clinical Decision-Making studied in geriatric emergency medicine

*Operationalization*	*Aid*	*Cognitive processes or contextual factors*	*Training or experience*
*CDM as dispositional decisions*	(6) (16) (41)	(8) (19) (21) (45) (51) (52)	(28) (42)
*CDM as cognition*	(32) (34)	(26) (33) (35) (30) (3) (29) (25**)** (46)	(13) (17) (15)
*CDM as a model*	(22) (39) (44) (7) (14) (48) (50)		
*CDM as clinical judgement*	(1) (11) (5) (18) (36) (4) (9) (20) (27) (40) (24) (37) (23) (2) (10) (12) (31) (38) (43) (47) (49)		

#### Effects of aids

When operationalizing CDM as clinical judgement (*n* = 21), the majority of studies [39, 43–54] investigating decision aids such as a tool, rule or standardized testing, found them to improve diagnostic accuracy and reduce uncertainty. However, an almost similarly large percentage found no difference [36, 42, 55, 56] or a decline in performance [57–60].

When CDM was operationalized as cognition, disposition decisions, or a model, studies overall found that an aid improved performance, in terms of decisions that are more accurate and lower uncertainty [21, 38, 40, 61–68].

#### Effects of cognitive processes or contextual factors

Studies aiming to discern the impact of cognitive behaviour such as confidence, heuristics, knowledge, skills or uncertainty or contextual factors such as practices or patient behaviour, predominantly operationalized CDM as dispositional decisions or cognitive processes. Regardless of the operationalization, the vast majority of studies [41, 69–78] found that clinician cognition or contextual factors negatively affected CDM performance or the accuracy of diagnostics. Only two [79, 80] found that CDM performance was unaffected by cognitive factors, however still arguing that this might not be true with more complex diseases [79]. This might explain why Seuren et al. [81] found that organizational structures like formalized multidisciplinary team meetings improved CDM practices.

#### The effects of training or experience

All studies investigating the impact of training or experience found that, regardless of whether CDM was operationalized as either cognitive processes [82–84] or dispositional decisions [85, 86], experience and reflective learning had a positive impact on the clinicians’ confidence, effectively improving skills, and possibly leading to more accurate decisions.

### CDM in GEM as a phenomenon

Finally, we synthesized how the included publications defined CDM, in order to describe some common conceptualizations and if, and how, they related those to GEM. As a large portion of publications (*n* = 39) did not provide a definition or description, the synthesis includes the thirteen studies which did, as presented in Table 4.

**Table 4:** Definitions and descriptions of Clinical Decision-Making in geriatric emergency medicine

***ID***	***Definition***	***CDM in GEM***	***Summary/themes***
*13*	“In making [treatment decisions, physicians] consider the disease, patient circumstances, and patient perceptions, as well as other factors. […] physicians engage in a large amount of mental processing [and] are often constrained by bounded rationality and satisficing...” (P154–155)	They found that the amount of treatment alternatives when encountering geriatric patients could alter decision-making. However, experience and the opportunity to supervise students reduced the risk of cognitive biases.	BIASES and DIAGNOSTICS Complexity in geriatric patients increases risk of bias, as it requires a larger amount of mental processing. REFLECTION Reflection helps.
*29*	““Clinical experience” consists of several components: [e.g.] accumulated knowledge [and] skill in collecting historical data... Knowledge is accumulated more or less [as a] data bank. Biases of availability, representativeness, and anchoring have been shown to be relevant, but it is not clear how much they detract from the value of “experience”.” (P163)	investigating coronary heart disease (CHD), which is a common geriatric medical condition, but does not address geriatric patients directly	BIASES Address a common geriatric disease. EXPERIENCE They found no effect of experience on decision making competencies in differential diagnosis of common conditions.
*30*	“… the ways in which the cognitive processes were used to solve the clinical problem had an enormous impact on the diagnostic error. The overreliance on the use of patterns was crucial.” (P1280)	Not addressed directly, other than the case description	DIAGNOSTIC ERROR: Overreliance on pattern-use in complex patients can increase diagnostic errors, and that errors are more likely to occur “...when clinical patterns run counter to expectations... [and that this] had a major role in causing the errors, rather than factors related to procedures or organization.”
*32*	Mental Shortcuts: “Cognitive errors are particularly frequent when the clinical decision-making process heavily relies on heuristics. These could be defined as ‘mental shortcuts’ …” (P2030). “Cognitive faculties deserve particular attention, as they are the bases of the clinical decision-making process … human abilities are limited and both gathering and retrieving information are inaccurate processes...”	Cognitive errors with geriatric patients because of failed heuristics and complexity with patients. Aiming to show how technology use can be a reliant tool.	HEURISTICS Complexity in patients cause errors in cognition, as it is guided by heuristics. Especially geriatric patients are complex REFLECTION “...continuous reappraisal and critical interpretation of all information are the mainstay of both the diagnosing process and the conscious use of heuristics.”
*35*	Builds upon several theories but concludes by formulating a model, which “...recognizes the salience of individual cognition, as well as acknowledging that the knowledge and experience that guides that cognition is constructed through social interaction and organizational context.” (P161)	“A number of studies internationally have identified that pain is often substantially undertreated or untreated in geriatric patients … There are particular issues with the management of pain for older patients in acute hospital settings.” (P153) “It moves beyond a model of pain recognition, assessment and management as being located within a sequential linear decision making framework, recognizing the importance of collaborative, co-constructed knowledge which develops time.” (P161)	COMMUNICATION It points to the importance of communication and patient involvement, especially with geriatric patients, in correct diagnostic assessment of pain.
*26*	Describes decision making as ‘mental models’ which is further described as thought processes. It refers to former studies describing “… how norms might affect hospital-based physician’s decision-making heuristics, case perceptions, and the consequential diagnosis and treatment...” (P345)	patient shared decision making/preferences and other situational characteristics influencing acute care decisions: Describing the physician’s mental models when encountering a terminally ill elderly patient and their decision to intubate or not and compare these with the appropriateness of the treatment plan (if the decision was a mistake or not). Treatment mistakes were related to patients reluctant to disclose mistakes to the physician and the physician reluctant to disclose uncertainty to patients.	DIAGNOSTIC ERROR Transparency between physician and patient affects risk of errors, but this was not compared between elderly and non-elderly patients. BIASES However, it was described that this transparency might be influenced by heuristics and social factors.
*33*	“… judgements are not based solely on a static phenomonen of pre-existing patient criteria, but come to be revised as the performance is played out throughout the interaction.” (P2449)	based on a geriatric clinical encounter, the authors note that “… the nurse possesses prior expectations as to how someone of this age would appear.” (P1446)	HEURISTICS Appraisal of the patient’s symptoms is guided by clinician heuristics, which result in over- or under-triage with geriatric patients
*17*	“Heuristics are mental shortcuts that often produce valid judgements but can lead to errors in atypical or rare events. Because they reflect natural processes, heuristics are not easily, or even productively, replaced.” (P9204)	No mention	HEURISTICS and EDUCATION They found that a narrative simulation game intervention reduced undertriage, by ‘recalibrating’ heuristics. This could be a result of the emotional part of a narrative approach, making them reflect upon their triage in another way. They did not, however, compare non-geriatric with geriatric patients, as all cases were geriatric, based on the assumption about common heuristics with elderly patients.
*19*	“The decision to order physical restraint is complex, influenced not only by the uncertainty resulting from lack of clinical guidelines and evidence, but also by organizational and situational factors and patient-specific variables. […] judgements are based on interactions between the environment and the individual.” (P1280)	“… lack of education regarding acute care geriatric medicine and physical restraint …” + “Presence of dementia increased the likelihood of having a restraint order 1.7 times. Very old age (85 years) resulted in a trend for lower likelihood …” (P1285)	EDUCATION A lack of geriatric knowledge in acute settings increases risk of treatment errors COMMUNICATION The presence of dementia increased risk of treatment errors due to poorer communication opportunities and increased complexity HEURISTIC Older age decreased risk of treatment errors as a result of frailty heuristics, which was unique for geriatric patients.
*34*	“Clinicians also use heuristic observation of objective factors and application of scientific data, but also ‘tacit’ knowledge based on acquired expertise and pattern recognition” (P116)	“The most important determinants of perception of inappropriate CPR were objective criteria such as … older age …” (P116)	HEURISTICS Older age increased risk of treatment errors in regards to CPR
*24*	“Clinical judgement can be defines as “an interpretation or conclusion about a patient’s needs, concerns, or health problems, and/or the decision to take action (or not), use or modify standard approaches, or improvise new ones as deemed appropriate by the patient’s response” [11]. It is complex and requires a flexible ability to recognise prominent aspects of an undefined clinical situation interpret their meaning and respond appropriately. It relates to the experience of individual clinicians.” (P5)	older patients are more often at high risk and current identification of these often relies on clinical judgement, which is flawed. Because of the complexity of these patients, a need for standardized, routine measurements are needed, in order to aid the identification of older patients at high risk of poor healthcare outcomes or admission to hospital.	TREATMENT ERRORS Elders are complex and therefore unaided clinical judgement alone is not enough. We need standardized measures to decrease risk of errors due to implicit flaws in cognition
***46***	“… at the individual level, we observed that ED physicians had the autonomy in decision-making [but] were also uncertainty avoidant when presented with equivocal results … At the ED-specific organisational level, this study highlighted the deep-rooted culture of the ED of practicing evidence-based Medicine [and how s] enior physicians were sources of information and role models … [P]hysician’s decision to prescribe antibiotics was [also] influenced at the community level by patient expectations” (P5–6)	Majority of the participants reported a lower threshold in prescribing antibiotics for elderly patients, especially those with comorbidities or were immunocompromised. The main reasons were to prevent any potential deterioration of the patient’s illness or occurrence of secondary bacterial infections. The availability of social support for elderly patients was also taken into consideration	HEURISTICS Heuristics about elderly patient’s frailty influenced prescription and the underuse of antibiotics amongst elderly patients UNCERTAINTY Physicians were uncertainty avoidant and tended to overprescribe antibiotics when faced with uncertainty
***52***	“Framing bias occurs when people make a decision based on the way the information is presented, as opposed to just on the facts themselves.” (P589)	No mention	BIAS How a case is framed has significant effect on differential diagnosis DIAGNOSTIC ERRORS These biases lead to diagnostic errors. However, it is still unclear is debiasing can prevent this

Of these thirteen publications, all but two [43, 78] operationalized CDM as cognition, describing the process itself and influencing factors. CDM was defined as a ‘mental process’ [21, 69, 82] referring to thought processes, which were complex and flexible abilities reflecting the individual’s knowledge and experience [28]. CDM as cognition was described as a pattern-recognising process [29], limited by cognitive retrieval [21, 43], and moderated by heuristics and biases [21, 64, 69, 70, 74, 77–79, 82, 83], social interaction and organizational context [41, 69, 70, 74, 77].

Overall, CDM with geriatric patients could be defined within four themes, commonly known in the CDM research, described below.

#### Diagnostic or treatment errors

The most prominent theme throughout all publications was clinicians’ risk of making errors in CDM. This was described as a result of overreliance on pattern-use [43], as more errors occurred when clinical findings conflicted with expectations, than as a result of inadequate clinical procedures or injudicious organizational factors [71]. Furthermore, errors were mediated by contextual factors such as social desirability [69, 78] i.e. when physicians were reluctant to disclose uncertainty.

#### Biases and heuristics

The included publications also linked increased error-risk to age-specific biases or overreliance on heuristics [69, 78, 79, 83]. Here, specifically the complexity of elderly patients were described to cause errors as normal clinical practice is guided by heuristics, which run the risk of simplifying complexity in urgent clinical settings [21]. As such, it was argued that CDM with elderly patients in EDs required a larger amount of mental processing [82]. In example, Edwards and Sines [70] described how the appraisal of symptoms was inherently guided by the clinician’s heuristics, resulting in over- or under-triage amongst elderly patients. However results varied, as some described how a frailty heuristic reduced risk of improper restraint orders [74], while others showed how they more often induced uncertainty, increasing risks in treatment decisions (i.e. ordering CPR [64] or prescribing antibiotics [77] and differential diagnosis [78].

#### Communication

Because of age-biased heuristics, one study underlined the importance of communication and patient involvement, especially with geriatric patients, when correctly diagnosing pain levels [41]. Here, the presence of dementia increased risk of treatment errors due to poorer communication opportunities and increased complexity [74].

#### Experience, education, and reflection

In order to counter these age effects on heuristics, and frailty biases, four studies addressed the impact of experience, education, and reflection. The studies found that a lack of geriatric knowledge in acute settings increases risk of treatment errors [74], but that reflection could help reduce the risk of cognitive biases [82]. Mohan, et al. [83] investigated the impact of different approaches to reflection and found that a narrative simulation game reduced under-triage, by ‘recalibrating’ heuristics. However, Fasoli, Lucchelli [79] argued that bias reduction interventions were ineffective with common diseases, emphasizing how complexity is a key factor when describing how and when errors occur.

### Summary of results

Overall, four overarching themes of operationalization of CDM emerged from the analysis. These operationalizations revealed different approaches to how clinical decisions in GEM settings are made. Some approached CDM as a cognitive phenomenon, or was concerned with different types of decision aids. However, the term ‘decision-making’ held some challenges by being a common phrase. Moreover, the theme ‘Clinical Judgement’ was commonly used as a delineated term, but most often referred to ‘practice as usual’, without relation to decision-making literature.

The different kinds of operationalizations led to various conclusions. When approaching decision-making as a cognitive process and looking at how decisions were made in practice, most found contextual or cognitive factors that influenced this process. However, when looking at decision-making as clinical judgement most studies were looking at how to aid routine judgement. Here, most studies found a positive impact on outcome measures, but contradictory results, might reflect the lack of homogeneity in how CDM were operationalized and measured.

It was evident how the complexity of geriatric patients held major challenges for CDM, and that e.g. frailty biases were commonly described to influence CDM competencies. However, it was also described how education and reflective practice could counter some of these effects.

## Discussion

This review set out to describe the concept of CDM in empirical research performed with elderly patients attending the ED. The aim was to provide a deeper understanding of the concept of CDM in this specific patient group and setting.

As described in the beginning of this paper, CDM is a vast field of research, drawing on several traditions from computational strategies and cognitive training, to sociology. However, recent discussions of this literature suggest that the field has moved away from a concept of the mind, focusing on error reduction, fragmented from the original theoretical assumptions [18, 87]. The result of this shift in the empirical investigation of CDM is that the exploration and intervention development become devoid of a unified theoretical framework. Moving research in such a direction could result in the development of interventions, which does not have the desired outcomes. The consequence of this lack of a theoretical framework was mirrored in prior reviews [10, 12, 14–16], which showed how difficult it was to synthesize this field of research. This review aimed to add insight into the challenges that we face and guide future research in the development and implementation of a concept of CDM.

### How was the theory of CDM reflected in the reviewed studies?

We looked at how studies within the included publications operationalized CDM and found that the majority of publications did not provide a theoretical framework for CDM. This led to notable differences in study objectives, which demonstrated this lack of consensus. Furthermore, a large number of the studies used decisions as a proxy measure, similarly not describing CDM within a theoretical framework. Here, the lack of a conceptualisation of the common term ‘clinical judgement’ lead to a methodological and ultimately, an empirical problem. With a under-defined and -described phenomenon, the operationalization risks being sporadic and unsystematic. Although the majority of studies found that decision aids had an impact on clinical judgement, the large amount of conflicting results points to problems with determining, which factors are causing different outcomes.

### What constitutes CDM in the context of GEM?

A synthesis of the eleven studies which provided a description or definition of CDM showed that the concept was generally understood as a cognitive process, affected by individual and contextual factors. Negative effects hereof were commonly countered by training or experience, pointing to emotional factors in reflection exercises, as more effective at prompting positive changes [83].

Geriatric patients were described as a particular complex patient-group, and a general frailty heuristic had an impact on CDM in different ways. It showed how this heuristic were both a protective factor in providing one treatment [74], and a risk factor providing another [21, 70], and that ‘recalibration’ of heuristics might be a trainable way of reducing errors [88]. Moving beyond the specific types of procedures, treatments or diagnosis, it could be relevant to know how the setting implicates such a frailty heuristic. This argument has also recently been highlighted by Woo [89] in their discussion on the coming challenges of the ageing population and the impact of contextual factors. This calls for investigations into how settings moderate and mediate proficient cognitive strategies, and how the interplay between cognition and context impose risks for the elderly patient, rather than polypharmacy or comorbidity in itself.

### Strengths and limitations

Focusing on a subarea of this body of literature is in itself narrowing the scope of the review, and its application to other domains. A narrow scope and a more theoretical analysis were necessary methodological compromises in this scoping review, focusing on other aspects of the reviewed studies may have yielded other perspectives. However, a systematic approach aimed to provide empirically founded arguments, and this can hopefully help qualify future research on CDM.

## Conclusion

In this scoping review we identified 52 studies addressing clinical decision-making for geriatric patients in emergency medical settings, published between 1981 and 2019. We aimed to clarify how a clinical decision was defined and operationalized. No systematic review had to our knowledge, explored the conceptual dimensions of CDM in the domain of GEM. Therefore, this scoping review set out to systematically analyze the definitions and operationalizations of CDM in empirical publications in GEM. We found that the majority of articles in this field of research did not provide a clear description or definition of the concept of CDM, and that the ones who did, primarily described it in cognitive terms. Only few studies pointed at contextual factors, arguing that CDM was, in fact, influenced by contextual or cognitive factors, when clinicians engage in complex decision-making. Age-specific biases were found to impact CDM in elderly patients in the ED, leading to errors in treatment and diagnosis. This was, however, not true in all circumstances, pointing to the importance of training of CDM competencies. However, as most of the included studies did not define CDM, it was not possible to formulate a clear conceptualization of CDM in GEM. Thus, such a conceptualizing may be the next step for future research. 

### Future research

Amongst the thirteen publications which provided atheoretical definition, some put emphasis on contextual factors impacting the cognitive CDM competency. This contextual component was, however, not a general theme in the reviewed publications and therefore needs to be elaborated on. As Hutchins [90] argue, we cannot meaningfully explore physician cognition as an isolated concept of the mind, as it is ontologically bound by the context of the ED, in which it operates. As such, there is a need for CDM studies focusing on the links between the cognitive components of the physician, and the contextual factors of the EM and interaction with geriatric patients.

In order to formulate a concept of CDM, it seems important to delve into some unanswered question in regards to what CDM is, and under which circumstances a competent clinical decision is mediated in the ED. We need to explore which parts of the process are inherent to, or learned by, the physician, and importantly, which elements are in fact not idiosyncratic but arise in the interaction with the context of the ED and geriatric patients.

## Supplementary information

**Additional file 1.** Study Characteristics.

**Additional file 2.** Coding of operationalizations of Clinical Decision-Making in geriatric emergency medicine.

## Data Availability

Not applicable.

## Abbreviations

CDM: Clinical Decision-Making
GEM: Geriatric Emergency Medicine
ED(s): Emergency Department(s)
EM: Emergency Medicine
PGY: Post Graduate Year (residents)
