# Supplementary material for: Conceptualizations of clinical decision-making: a scoping review in geriatric emergency medicine
Source: BMC Emerg Med. 2020 Sep 14;20:73. doi: 10.1186/s12873-020-00367-2 (PMC7489001; doi:10.1186/s12873-020-00367-2)
Supplement: Supplementary file 1 — Additional file 1. Study Characteristics. [file 12873_2020_367_MOESM1_ESM.doc]

***Appendix 1: Study Characteristics***

| **Author** | **Year** | **Title** | **Method** | **Aim** | **ED Setting** | **Outcome measure** | **Summary of results** | **ED-participants** | **Geriatric patient group** |
| --- | --- | --- | --- | --- | --- | --- | --- | --- | --- |
| van der Burg-de Graauw, N.; Cobbaert, C. M.; Middelhoff, C. J. F. M.; Bantje, T. A.; van Guldener, C. | 2009 | The additive value of N-terminal pro-B-type natriuretic peptide testing at the emergency department in patients with acute dyspnoea | Prospective observational study | To compare NT-proBNP and clinical judgement in diagnosing acute dyspnoea | N=1 | Clinical features, physicians's probability estimate of heart failure (0-100%) and clinical diagnosis, and final diagnosis by an independent interdisciplinary panel | "NT-proBNP measurement has additive value in a substantial number of patients presenting with acute dyspnoea, but can possibly be omitted in patients with a clinical probability of heat failure of <70%." (P301) | Physicians (no N) | Mean=71.1 (SD=14.3), N=221 |
| Visser, A.; Wolthuis, A.; Breedveld, R.; Ter Avest, E. | 2015 | HEART score and clinical gestalt have similar diagnostic accuracy for diagnosing ACS in an unselected population of patients with chest pain presenting in the ED | Prospective observational study with an 8-month follow-up | To compare the diagnostic accuracy of clinical judgement with the HEART score in acute coronary syndrome | N=1 (teaching hospital) | Case Report Form (all clinical features); physician’s clinical gestalt (probability of ACS; low, intermediate,high), HEART score | "…HEART score and clinical gestalt have similar diagnostic accuracy for diagnosic ACS in an unselected population of patients with chest pain presenting in the ED." (P595) | Physicians | M=69 (subgroup of patients >65 years: N=120) |
| Viswanathan, K.; Rosen, T.; Mulcare, M. R.; Clark, S.; Hayes, J.; Lachs, M. S.; Flomenbaum, N. E. | 2015 | Emergency Department Placement and Management of Indwelling Urinary Catheters in Older Adults: Knowledge, Attitudes, and Practice | Survey study (N=129) and case study (N=25) | To explore ED personnel’s knowledge, attitudes, and practice in decision-making about UIC intervention in older adults | N=1 (large urban, academic medical center) | Incorporated items from previously published instruments to measure knowledge, attitudes and practice in a written survey, measured through Likert scales | "Wide practice variation exists between individual providers [despite] emergency nurses and other providers report comfort with appropriate conditions for UIC placement…" (P414) | Nurses (N=43), attending physicians (N=20), midlevel providers (N=19), and resident physicians (N=47) | Unknown, (focused on elderly patients) |
| Vora, N.; Tung, C. E.; Mlynash, M.; Garcia, M.; Kemp, S.; Kleinman, J.; Zaharchuk, G.; Albers, G.; Olivot, J. M. | 2015 | TIA triage in emergency department using acute MRI (TIA-TEAM): A feasibility and safety study | Intervention study, with follow-up at 7, 90 and 365 days | To evaluate the feasibility of an MRI-based triage system in a clinical decision unit | N=1 | TIA index and occurrences of stroke, MI or death. Clinical judgement=likelihood assessment of risk outcomes | "TIA traige and acute MRI is feasible and safe." (P343) | Specialist physicians | M=69 (SD=17), N=129 |
| Wallgren, U. M.; Antonsson, V. E.; Castrén, M. K.; Kurl; , L. | 2016 | Longer time to antibiotics and higher mortality among septic patients with non-specific presentations -a cross sectional study of Emergency Department patients indicating that a screening tool may improve identification | Retrospective cross-sectional observational study | To compare efficacy of a screening tool with clinical judgement in diagnosing septic patients with non-specific presentation | N=1 | Time to administration of antibiotics, in-hospital mortality, Robson screening tool for sepsis, and documentation of the clinical judgement of the presence and severity of sepsis | "…implementation of a screening tool may increase the identification of septic patients." (P1) | ED physicians (no N) | Intervention group: N=61, m=78 (67-85); sepsis reference group: N=516, m=73 (61-82) |
| Young, G. P.; Hedges, J. R.; Brian Gibler, W.; Green, T. R.; Swanson, R. | 1991 | Do CK-MB results affect chest pain decision making in the emergency department? | Intervention study | To describe how diagnostic hypothesis can be improved by different diagnostic information | N=2 (teaching hospitals) | Probability measure of AMI with or without CK-MB knowledge | Real-time provision of CK-MB results were helpful, especially in the geriatric subgroup, in the decision-making process, leading to lower uncertainty, however uncertainty is not | Resident physicians (PGY 1-3) | N = 12 (M=65,3) |
| Suffoletto, B.; Miller, T.; Frisch, A.; Callaway, C. | 2013 | Emergency physician recognition of delirium | Prospective, observational study | To compare clinical judgement with a decision-making tool (CAM) in the diagnosis of delirium | N=2 | Rating of delirium (CAM criteria) | "When emergency physicians use routine clinical observations, they may miss diagnosing up to two-thirds of patients with delirium. Recognition of  delirium can be enhanced with standardised cognitive  testing." (P621) | ED physicians (no N) | N=302 patients >65 years (mean=80, SD=8) |
| Stoklosa, H.; Scannell, M.; Ma, Z.; Rosner, B.; Hughes, A.; Bohan, J. S. | 2018 | Do EPs change their clinical behaviour in the hallway or when a companion is present? A cross-sectional survey | Cross-sectional survey | Evaluating the impact of disruption by clinical staff on diagnostic decision-making in doctor-patient encounters | N=1 | diagnostic error | "Our study suggests that alterations in EP usual practice occurs when the doctor-patient dyad is disrupted by evaluation in a hallway or presence of a companion [which] are associated with delays in car and failure to diagnosis medical, social and psychiatric conditions." (P406) | Physicians | Only mentions 'elder abuse' |
| Stevenson, J.; Parekh, N.; Ali, K.; Timeyin, J.; Bremner, S.; Van Der Cammen, T.; Allen, J.; Schiff, R.; Harchowal, J.; Davies, G.; Rajkumar, C. | 2016 | Protocol for a Prospective (P) study to develop a model to stratify the risk (RI) of medication (M) related harm in hospitalized elderly (E) patients in the UK (The PRIME study) | Prospective observational study, with an 8-week follow-up. n | To (develop and) compare the efficacy of a risk prediction model to clinical judgement in identifying elders at high risk of medication related harm upon discharge from hospital | N=1 (in-patient wards) | Patient data, likelihood judgement of MRH (4-point scale) and confidence in this judgement (6-point scale) | Not applicable (not yet finalized) | Junior doctors | Patients >65 years |
| So, S. N.; Ong, C. W.; Wong, L. Y.; Chung, J. Y.; Graham, C. A. | 2015 | Is the Modified Early Warning Score able to enhance clinical observation to detect deteriorating patients earlier in an Accident & Emergency Department? | Observational study | To compare clinical judgement with a decision aid (Modified Early Warning Scare) | N=1 | Patient outcomes (deterioration) | "Using the MEWS for patient monitoring did not significantly enhance the performance in detecting patient deterioration for a group of patients who are waiting for in-patient beds in a public ED." (P25) | Nurses | MEWS group: N=269 (M=71.6), CJ group: N=275 (M=70.8) |
| Sirois, M. J.; Griffith, L.; Perry, J.; Daoust, R.; Veillette, N.; Lee, J.; Pelletier, M.; Wilding, L.; Émond, M. | 2017 | Measuring frailty can help emergency departments identify independent seniors at risk of functional decline after minor injuries | Prospective observational study | To describe frailty amongst elderly and compare clinical judgement with a risk screening tool for functional decline | Non-defined | Frailty (CSHA-CFS and SOF), functional decline (Older American Recourses Services scale), Clinical Judgement (no mention) | "Measuring frailty in community-dwelling seniors with minor injuries in EDs may enhance current risk screening for functional decline." (P68) | Physicians and nurses recruited participants (no N) | N=1072 (M=77.1, ±7,5 years) |
| Seymann, G.; Barger, K.; Choo, S.; Sawhney, S.; Davis, D. | 2008 | Clinical Judgment versus the Pneumonia Severity Index in Making the Admission Decision | Retrospective observational study | To compare clinical judgement with a decision aid (PSI screening) in triage decisions | N=1 (teaching hospital) | PSI scores (retrospective chart review), clinical judgement: patient outcomes | "Low-risk inpatients had a significant length of stay, suggesting that clinical judgment appropriately superseded the PSI in these cases." (P261) | Non-specified | N=174, no M age, (geriatric patients in the high-risk subgroup) |
| Roswarski, T. E.; Murray, M. D. | 2006 | Supervision of students may protect academic physicians from cognitive bias: A study of decision making and multiple treatment alternatives in medicine | Experimental survey study using case scenarios | To describe the impact of physician’s characteristics and practices on their decision-making | N=1 (medical school department) | Physician’s treatment decisions | Multiple treatment alternatives might lead to deferred decisions. Supervising students were less likely to be affected by cognitive biases when presented with more than one treatment option. | Residents, fellows, faculty and staff physicians (N=192) | Case scenario: 67-year-old patient |
| Ranzani, O. T.; Prina, E.; Menendez, R.; Ceccato, A.; Cilloniz, C.; Mendez, R.; Gabarrus, A.; Barbeta, E.; Bassi, G. L.; Ferrer, M.; Torres, A. | 2017 | New Sepsis Definition (Sepsis-3) and Community-acquired Pneumonia Mortality. A Validation and Clinical Decision-Making Study | Cohort study | To use decision-analysis to evaluate three decision-making tools (SIRS, qSOFA, and CRB) compared to clinical judgment in identifying sepsis | N=2 (community hospitals) | clinical features, treatments performed, Sepsis-3 flowchart and several tools for identification of sepsis, measuring sensitivity and specificity | "qSOFA and CRB outperformed SIRS and presented better clinical usefulness as prompt tools for patients with community-acquired pneumonia in the emergency department. Among the tools for a comprehensive patient assessment, PSI had the best decision-aid tool profile." (P1287) | unknown | M=66.1 (SD=19) |
| Prendergast, H. M.; Jurivich, D.; Edison, M.; Bunney, E. B.; Williams, J.; Schlichting, A. | 2010 | Preparing the Front Line for the Increase in the Aging Population: Geriatric Curriculum Development for an Emergency Medicine Residency Program | Pre- and post-intervention survey study | To describe the impact of an geriatric educational program on ED resident’s knowledge and attitudes towards elderly patients | Non-defined | Geriatric attitude scale survey | "Using familiar educational formats with heavy emphasis on "hands-on" activities to present the geriatric care curriculum had a positive impact on resident knowledge and confidence in dealing with geriatric patients." (P386) | Emergency Medicine Residents (PGY1-3) | Geriatric curriculum (no age) |
| Politi, L.; Codish, S.; Sagy, I.; Fink, L. | 2015 | Use patterns of health information exchange systems and admission decisions: Reductionistic and configurational approaches | Retrospective observational study | To explore the impact of Health Information Exchange systems as a decision-aid on clinical decision-making | N=1 | Observational data of health information exchange (HIE) use and decisions to admit | "The study shows that congruent profiles of HIE use enhance the predictability of the admission decision" (P1029); "Moreover, these findings confirm the fundamental importance of information availability to reducing uncertainty, which is inherent to the clinical decision making process…" (P1035) | ED physicians (no N) | M=68.79 (SD=16.34) |
| Mohan, D.; Fischhoff, B.; Angus, D. C.; Rosengart, M. R.; Wallace, D. J.; Yealy, D. M.; Farris, C.; Chang, C. C. H.; Kerti, S.; Barnato, A. E. | 2018 | Serious games may improve physician heuristics in trauma triage | Intervention study: RCT | To explore the impact of two interventions to improve physician’s heuristic (a game and a text-based) on triage accuracy | No setting (only ED physicians) | Performance (N=under-triaged patients), enjoyment (5-point Likert scale) and a narrative engagement scale. | "We found that both game interventions reduced undertriage in the simulation, compared with the control condition, whereas the text-based intervention did not." (P9207) | Physicians (N=320) | 6 geriatric cases in the virtual simulation |
| Moe, G. W.; Howlett, J.; Januzzi, J. L.; Zowall, H. | 2007 | N-terminal pro-B-type natriuretic peptide testing improves the management of patients with suspected acute heart failure: Primary results of the Canadian prospective randomized multicenter IMPROVE-CHF study | Randomized, controlled, double-blind, prospective multicenter study | To compare the accuracy of clinical judgement with and without a diagnostic information (NT-proBNP) in diagnosing heart failure | N=7 | Clinical features and ED physician's diagnosis of the presence of HF (no/yes) and likelihood of the presence of acute HF was the cause of dyspnoea (0-100%) | "NT-proBNP testing improves the management of patients presenting to emergency departments with dyspnoea through improved diagnosis, cost savings, and improvement in selected outcomes." (P3103) | ED physicians (no N) | Intervention: N=264 (M=70, SD=15); Usual care: N=254 (M=71, SD=14) |
| Mion, L. C.; S; hu, S. K.; Khan, R. H.; Ludwick, R.; Claridge, J. A.; Pile, J.; Harrington, M.; Dietrich, M. S.; Winchell, J. | 2010 | Effect of Situational and Clinical Variables on the Likelihood of Physicians Ordering Physical Restraints | Cross-sectional, factorial survey | "…to identify factors influencing physician's likelihood to order physical restraints." (P1280) | N=1 | Likelihood of ordering restraint (10-point scale) | Unsafe patient behaviour and dementia increased the likelihood of ordering restraint, whereas lack of trust in the reporting nurse decreased the likelihood of ordering restraint. Clinical factors play a lesser part compared to working relations and patient behaviour when physicians are considering ordering restraint | Physicians (N=189) from other specialties, solving an acute care case vignette | Geriatric case vignettes (65 and 85) |
| McCullough, P. A.; Nowak, R. M.; McCord, J.; Holl; er, J. E.; Herrmann, H. C.; Steg, P. G. | 2002 | B-type natriuretic peptide and clinical judgment in emergency diagnosis of heart failure: Analysis from Breathing Not Properly (BNP) Multinational Study | Prospective observational study | To compare the accuracy of clinical judgement with and without a diagnostic information (B-type peptide) in diagnosing congestive heart failure. | N=7 (US=5, EU=2) | B-type Natriuretic Peptide, clinical judgement: routine un-aided judgement. | "The evaluation of acute dyspnoea would be improved with the addition of BNP testing to clinical judgement in the emergency department." (P416) | Physicians | 2 geriatric subgroups: intermediate probability (M=67.8, ±14.6), high probability (M=70.23, ±14.8) |
| Lewis, L. M.; Klippel, A. P.; Bavolek, R. A.; Ross, L. M.; Scherer, T. M.; Banet, G. A. | 2007 | Quantifying the usefulness of CT in evaluating seniors with abdominal pain | Prospective observational study | To describe the impact information (CT) on diagnostic accuracy in evaluating abdominal pain, and which factors influenced the decision to order a CT | N=1 (teaching hospital) | Diagnosis and confidence levels, with retrospective reflections upon the physician’s decision-making | Lower confidence more often lead to order a CT, which then more often resulted in a change in diagnosis, which increased confidence | Physicians (no N) | Stratified ages: 60-74 (N=75) and >74 (N=51) |
| Jessen, M. K.; Mackenhauer, J.; Hvass, A. M. W.; Ellermann-Eriksen, S.; Skibsted, S.; Kirkegaard, H.; et al. | 2016 | Prediction of bacteraemia in the emergency department: An external validation of a clinical decision rule | retrospective cohort study | To validate an infection clinical decision rule, by its diagnostic accuracy | N=1 | "true bacteraemia" (fungal or bacterial growth in blood cultures) | "The clinical decision rule performed well in our ED setting and is likely to be a useful supplement to clinical judgment." (P44) | not applicable | Bacteraemia subgroup: M=71.2 (SD=17) |
| Januzzi Jr, J. L.; Camargo, C. A.; Anwaruddin, S.; Baggish, A. L.; Chen, A. A.; Krauser, D. G.; et al. | 2005 | The N-terminal Pro-BNP investigation of dyspnoea in the emergency department (PRIDE) study | Prospective observational study with a 60-day follow-up | To compare the accuracy of clinical judgement with and without a diagnostic information (NT-phoBNP) in diagnosing congestive heart failure | N=1 | Dispositions decisions of final diagnosis of acute CHF | "NT-proBNP plus clinical judgement was superior to NT-proBNP or clinical judgement alone." (P948) | Specialist Physicians (cardiologists, no N) | Acute CHF patient subgroup, N=209 (M= 72.8, SD= 13.6) |
| Ibrahim, K.; Owen, C.; Patel, H. P.; May, C.; Baxter, M.; Sayer, A. A.; Roberts, H. C. | 2017 | Can routine clinical data identify older patients at risk of poor healthcare outcomes on admission to hospital? | Interview and focus group study, and statistical review of 60 patients' clinical records to identify characteristics | To identify current practices involved in risk-identification of elderly at high risk for adverse health outcomes. | N=5 | Participant reports on the use of clinical data, compared to patient characteristics and outcomes. | “Staff relied on their clinical judgement to identify high risk patients… A number of risk assessments were carried out routinely but used mainly to identify single healthcare needs, rather than holistically to identify individuals at risk of poor healthcare outcomes…” (P5) | Multidisciplinary healthcare staff (N=22): nurses, dieticians, therapists, physician consultants, residents (PGY1-2), specialist physicians | N=60, (M= 86.7, SD=5.3) |
| Hillinger, P.; Twerenbold, R.; Wildi, K.; Rubini Gimenez, M.; Jaeger, C.; Boeddinghaus, J.; et al. | 2017 | Gender-specific uncertainties in the diagnosis of acute coronary syndrome | prospective, observational study | To discern the impact of delayed and misdiagnosis of acute coronary syndrome in women on adverse outcomes | N=9 (3 countries) | Clinical judgement: VAS scale for ACS probability (0-100%) Late diagnostic uncertainty: "...disagreement among two independent cardiologists' adjudication of the final diagnosis after complete work-up." (P28) | They did not find a coherence with diagnostic uncertainty and poorer outcome for women. | Treating ED physicians (no N) | m=62 years (50-75) |
| Haliko, S.; Downs, J.; Mohan, D.; Arnold, R.; Barnato, A. E. | 2018 | Hospital-Based Physicians' Intubation Decisions and Associated Mental Models when Managing a Critically and Terminally Ill Older Patient | Retrospective think aloud study on a simulated patient encounter | To describe physician mental models when encountering terminally ill elderly patients and its impact on the decision to intubate or not, and its appropriateness. | No setting (ED case material) | Mental models (concept mapping, semantic web and decision analysis) and appropriateness of treatment plans (level of patient preference concordance, compared to expert models by statistical regression analysis) | Treatment mistakes were related to patients reluctant to disclose mistakes to the physician and the physician reluctant to disclose uncertainty to patients. Three factors influences the decision to intubate or not: (1) counting in terminal diagnosis in considering patient’s treatment preferences (2) physician's ability to be objective and ignore discomfort with terminal diagnosis, and (3) the weight given to patient's treatment preferences | Physicians (N=73) | simulated case material: late 70s |
| Green, S. M.; Martinez-Rumayor, A.; Gregory, S. A.; Baggish, A. L.; O'Donoghue, M. L.; Green, J. A.; Lew; rowski, K. B.; Januzzi Jr, J. L. | 2008 | Clinical uncertainty, diagnostic accuracy, and outcomes in emergency department patients presenting with dyspnoea | Retrospective observational study | To explore uncertainty related to adverse events and the impact of diagnostic information (NT-proBNP) on clinical judgement | N=1 | clinical features, probability rates of likelihood for ADHF (0-100%) and a certainty score (0-100%) | "Among dyspnoeic patients in the ED, clinical uncertainty is associated with increased morbidity and mortality, especially in those with ADHF. The addition of NT-proBNP testing to clinical judgement may reduce diagnostic uncertainty in this setting." (P741) | Physicians | Subgroup of ‘clinical uncertainty’: M=69 (SD=14) |
| Fry, M.; Macgregor, C.; Hyl; , S.; Payne, B.; Chenoweth, L.; | 2015 | Emergency nurses' perceptions of the role of confidence, self-efficacy and reflexivity in managing the cognitively impaired older person in pain | focus group interviews (N=16) | To explore the impact of confidence and self-efficacy on the effectiveness of clinical care practice | N=4 | 15-item semi-structured interview tool: role of self-efficacy and reflectivity (open-ended questions), confidence (Likert-scale) | "Our findings demonstrate that confidence, self-efficacy and reflexivity enabled the delivery of appropriate, timely and compassionate care… [which] relied on clinical experience and reflective learning..." (P1622) | Nurses (N=80) | Defined as ‘older person with cognitive impairment’ |
| Fasoli, A.; Lucchelli, S.; Fasoli, R. | 1998 | The role of clinical 'experience' in diagnostic performance | Longitudinal study, with a five-year follow-up. | To describe the impact of knowledge and skill on clinical reasoning in regards to diagnostic accuracy. | No setting (ED case material) | Probability measure of giving a Coronary Heart Disease diagnosis based on Baye's rule, and then assessing diagnostic errors using ROC analysis | They found no effect of experience on decision making competencies in differential diagnosis of common conditions | Physicians (N=21) | 2 geriatric cases (stratified ages 65-74 and >74) |
| Famularo, G.; Salvini, P.; Terranova, A.; Gerace, C. | 2000 | Clinical errors in emergency medicine: Experience at the emergency department of an Italian teaching hospital | Case study (N=4) | To describe the relation between subjective interpretation and organizational factors on diagnostic errors | N=1 (teaching hospital) | Not Applicable | "Analysis suggested that the process of clinical decision making and the over-reliance on the use of patterns during the cognitive process had a major role in causing the erros, rather than factors related to procedures or organization." (P1278) | Emergency physicians (no N) | Geriatric cases: age 86 and 65. |
| Elliott, A.; Phelps, K.; Regen, E.; Conroy, S. P | 2017 | Identifying frailty in the Emergency Department-feasibility study | Mixed methods study: Focus group interviews and experimental intervention study | To compare clinical judgement with several risk-prediction tools in regards to physician’s experience with the tool. | N=1 (teaching hospital) | Identification of Seniors At Risk = Clinical Frailty Scale, PRISMA-7 and Silver Code; clinical judgement = unaided judgement | "There were no significant differences in timing, ease of use or agreement with clinical judgement between tools." (P840) | Nurses, and junior and senior doctors (N=121) | Geriatric case vignettes |
| Elia, F.; Panero, F.; Molino, P.; Ferrari, G.; Apra, F. | 2012 | Ultrasound to reduce cognitive errors in the ED | Case study (N=3) | To explore cognitive errors and the impact of debiasing strategies such as ultrasonography | N=1 | not Applicable | "...continuous reappraisal and critical interpretation of all information are the mainstay of both the diagnosing process and the conscious use of heuristics. Ultrasonography may deserve an important role in overcoming our cognitive limits as any other debiasing strategy. In the same way, its correct application requires adequate training, knowledge of its limitation and deliberate critic." (P2033) | Not specified | Geriatric cases: age 68 and 74 |
| Edwards, B.; Sines, D. | 2008 | Passing the audition - The appraisal of client credibility and assessment by nurses at triage | Retrospective think aloud study | To describe cognitive processes involved in initial triage decisions by nurses | N=2 | not applicable | Triage can be regarded as a process in which nurses act as an adjudicating panel, judging the clinical data by appraisal of patient behaviour | Emergency nurses (N=14) | Not specifically reported |
| Druwé, P.; Monsieurs, K. G.; Piers, R.; Gagg, J.; Nakahara, S.; Alpert, E. A.; et al. | 2018 | Perception of inappropriate cardiopulmonary resuscitation by clinicians working in emergency departments and ambulance services: The REAPPROPRIATE international, multi-centre, cross sectional survey | International, multi-centre, cross sectional survey study | To determine the prevalence of uncertainty in CPR decisions and its impact on adverse events, as well as exploring the relevance of patient- and situation-related factors in regards to appropriateness. | N=288 (24 countries) | A description of the clinician's last attempt at CPR, and their rating of appropriateness (Likert scale) related to certainty. | "...experienced clinicians who are uncertain about CPR appropriateness… deserve to be acknowledged [and factoring] this clinical judgement into the decision to attempt resuscitation may reduce harm… facilitating a more dignified death for many." (P119) | ED and prehospital clinicians (N=4123); doctors (N=1481), nurses (N=1009), and EMTs (N=1528) | No mean (geriatric subgroup older than 79) |
| Dowding, D.; Lichtner, V.; Allcock, N.; Briggs, M.; James, K.; Keady, J.; Lasrado, R.; Sampson, E. L.; Swarbrick, C.; José Closs, S. | 2016 | Using sense-making theory to aid understanding of the recognition, assessment and management of pain in patients with dementia in acute hospital settings | Ethnography | To conceptualize DM for pain management of patients with dementia in acute care settings | N=1 | Not applicable | DM in pain management of patients with dementia is a group process embedded in a specific culture, summarized in a cognitive model. 3 themes emerged: (1) as pain fluctuates, assessment of does as well, (2) the context of the ward affects how pain is recognised, assessed and managed, (3) pain is recognised, assessed and managed by many individuals over a long period of time, and is therefore a process of trial and error and team communication. | Physicians, staff nurses, charge nurses, ward managers, support workers and specialist nurses (N=52) | M=88 years (N=31) |
| Di Somma, S.; Magrini, L.; De Berardinis, B.; Marino, R.; Ferri, E.; Moscatelli, P.; Ballarino, P.; Carpinteri, G.; Noto, P.; Gliozzo, B.; Paladino, L.; Di Stasio, E. | 2013 | Additive value of blood neutrophil gelatinase-associated lipocalin to clinical judgement in acute kidney injury diagnosis and mortality prediction in patients hospitalized from the emergency department | Prospective cohort study | To compare clinical judgement with a risk-prediction tool (blood neuropil gelatinase associated lipocalin) in diagnosing acute kidney failure | N=3 | Anamnestic and diagnostic information, ED physician's initial clinical assessment (AKI or NO AKI), and level of confidence (0-100%) | "…assessment of patient's initial blood NGAL when admitted to hospital from the ED improved initial clinical diagnosis of AKI and predicted in-hospital mortality. [The] assessment coupled with the ED physician's clinical judgement may prove useful…" (P1) | ED physicians | M=74, SD=14.4 |
| De Groot, B.; Lameijer, J.; De Deckere, E. R. J. T.; Vis, A. | 2014 | The prognostic performance of the predisposition, infection, response and organ failure (PIRO) classification in high-risk and low-risk emergency department sepsis populations: Comparison with clinical judgement and sepsis category | Observational prospective study | To compare the prognostic value of two decision making tools (PIRO and MEDS) and clinical judgement between high- and low-risk patients in diagnosing sepsis | N=2 | disposition decisions, and evaluation scores based on the guidelines used | The PIRO score and clinical judgement perform similarly, with the risk-stratification tool proving more useful in high-risk populations. | ED physicians (No N) | M= 66 (SD=17) |
| de Groot, B.; de Deckere, E. R.; Flameling, R.; S; el, M. H.; Vis, A. | 2012 | Performance of illness severity scores to guide disposition of emergency department patients with severe sepsis or septic shock | prospective observational cohort study | To compare clinical judgement with PIRO in diagnosing sepsis. | N=2 | PIRO and MEDS scores, clinical judgement = the unaided decision; also measuring sensitivity and specificity | "The PIRO score adds little valure over clinical judgement in guiding adequate disposition to wards or the ICU." (316) | ED physicians (no N) | Several geriatric subgroups; only one not within inclusion criterias (ICU admitted patients) |
| Davey, K.; Saul, T.; Russel, G.; Wassermann, J.; Quaas, J. | 2018 | Application of the Canadian Computed Tomography Head Rule to Patients with Minimal Head Injury | prospective observational cohort study with a convenience sample of patients | To investigate physician’s motivations for ordering CT, and validating the accuracy of a decision-making rule (Canadian CT Head Rule) against clinical judgement in diagnosing head injury. | N=2 (teaching hospitals) | Head CT Rule form, and reports on physician's assessment of features of the history and physical examination that impacted the decision to order a CT | "Our study suggest that the Canadian CT Head Rule is an effective screening tool for patients with minimal head injury." (P349) | Physicians (attending and PGY3 residents) | geriatric subgroup of >65 (N=137) |
| Conti, A.; Poggioni, C.; Viviani, G.; Luzzi, M.; Vicidomini, S.; Zanobetti, M.; Innocenti, F.; Pini, R.; Padeletti, L.; Gensini, G. F. | 2012 | Short- and long-term cardiac events in patients with chest pain with or without known existing coronary disease presenting normal electrocardiogram | Prospective, nonrandomized study with follow-up at 1, 6 and 12 months | To evaluate the effect of using diagnostic information (electrocardiogram) as a risk stratification tool on adverse events, compared to clinical judgement in patients with coronary disease. | N=1 (teaching hospital) | Clinical features, end-point events | Risk stratification and stress testing was effective in patients without existing coronary disease. | Not specified | 1 geriatric subgroup: known coronary disease, M=72 (SD=12) |
| Cohn, S.; Fritz, Z. B. M.; Frankau, J. M.; Laroche, C. M.; Fuld, J. P. | 2013 | Do not attempt cardiopulmonary resuscitation orders in acute medical settings: A qualitative study | Observation and semi-structured interviews | To describe the use of DNACPR forms, and their impact on patient care decisions. | N=2 | Not Applicable | DNACPR orders can often signify inproper care and decision making, which makes clinicians uncomfortable with discussing these with patients and families | Ward staff (N=28, 13 physicians, and 14 nurses) | Not specified |
| Chipman, C.; Adelman, R.; Sexton, G. | 1981 | Criteria for cessation of CPR in the emergency department | Cross-sectional survey | To describe criteria to inform the decision to cease CPR, in order to explore possible general guidelines. | N=2 | Decision to cease CPR or not | Type of residency, city size, and ED experience (yeas) correlated with how the decision to cease CPR was made. | Physicians (N = 78) | Geriatric criteria for cessation of CPR |
| Caterino, J. M.; Leininger, R.; Kline, D. M.; Southerl; , L. T.; Khaliqdina, S.; Baugh, C. W.; Pallin, D. J.; Stevenson, K. B. | 2017 | Accuracy of Current Diagnostic Criteria for Acute Bacterial Infection in Older Adults in the Emergency Department | Prospective, observational study | To compare the accuracy of clinical judgement with a decision-making rule (Loeb criteria) in diagnosing sepsis | N=1 (Urban, tertiary-care) | Identification of bacterial infection with or without guidelines. Sensitivity and specificity reported | The Loeb criteria are useful only for diagnosing skin and soft tissue infections, CDC guidelines are inadequate in the ED. New criteria are needed | Emergency physicians (no N) | M= 74, SD=7.4 (N=424) |
| Brixner, D.; Biltaji, E.; Bress, A.; Unni, S.; Ye, X.; Mamiya, T.; Ashcraft, K.; Biskupiak, J. | 2015 | The effect of pharmacogenetic profiling with a clinical decision support tool on healthcare resource utilization and estimated costs in the elderly exposed to polypharmacy | prospective observational cohort study | To assess the effect of diagnostic information and a clinical decision support tool on the use of healthcare resources in regards to adverse events amongst patients with polypharmacy | No setting (ED visits) | Adverse events, YouScript use | "…CYP testing of the elderly exposed to polypharmacy, along with appropriate clinical decision support tools, such as YouSCript, may provide valuable information to guide prescription drug treatment, reduce hospitalization and ED visits, and lower overall costs." (P223) | 'Providers' | Tested: M=75 (SD=6.9); untested: M=75 (SD=6.5) |
| Pinkney, J.; Rance, S.; Benger, J.; Brant, H.; Joel-Edgar, S.; Swancutt, D.; et al. | 2016 | Health Services and Delivery Research | Multiple case study: ethnography and value stream mapping | To describe admission and discharge decisions and investigate the impact of expertise and models of care on clinical decision-making in regards to admission and discharge | N=4 | Participant observation (workflows, patient waiting times) and field interviews (professionals, patients and carers) | "Influences on decision-making included [time] pressure on staff […]; professionals’ ability to balance risk and safety; and patients’ home care situations…" (Pxix) | Nurses and physicians | Stratified ages: 65-74, 75-84 and ≥85 |
| Chan, Y. Y.; Bin Ibrahim, M. A.; Wong, C. M.; Ooi, C. K.; Chow, A. | 2019 | Determinants of antibiotic prescribing for upper respiratory tract infections in an emergency department with good primary care access: A qualitative analysis | Semi-structured interview study | To describe determinants of prescribing antibiotics to patients presenting with upper respiratory tract infections (URTI) in time-strapped ED settings | N=1 | Thematic analysis of determinants for prescribing antibiotics | “Organisational practice norms strongly influenced antibiotic prescribing decisions... Clinical decision support tools, hospital guidelines and patient education […] could reduce unnecessary antibiotic use.” (P1) | Physicians (N=9) | Elderly patients are mentioned specifically, reporting lower prescribing tendencies amongst this patient group. |
| Eagles, D.; Otal, D.; Wilding, L.; Sinha, S.; Thiruganasamb; amoorthy, V.; Wells, G. A.; Stiell, I. G. | 2019 | Evaluation of the Ottawa 3DY as a screening tool for cognitive impairment in older emergency department patients | Prospective cohort study | To evaluate the implementation of a screening tool for cognitive impairment, compared to clinical judgement. | N=1 | Descriptive survey data of implementation and acceptability and prognostic value of the tool | The tool was feasible to implement and added accuracy for evaluating cognitive impairment, compared to routine clinical judgement. | Nurses and physicians (no N) | M=83.7, SD= 5.9 (N=260) |
| Levinson, M.; Walker, K. J.; Hanning, J.; Dunlop, W.; Cheong, E.; Mills, A. | 2019 | Medical perspectives regarding goals-of-care consultations in Emergency Departments | Interview study | To explore ED providers’ perspectives in goals-of-care discussions regarding end-of-life decisions, as well as establish patient values for shared decision-making | N=1 | Themes amongst ED providers | “Emergency doctors perceive goals-of-care discussions to be relevant to their  Practice... They aim to ensure appropriate care is provided [and]  felt they could recognise end-of-life and that ED visits often prompt consideration of end-of-life care  planning. They wanted long-term practitioners to initiate discussions  prior to patient deterioration.” (P1137) | Physicians (N=18) | It is mentioned, that this group of patients are elderly, and that this particular ED setting primarily deal with older adults |
| Lewis, E. T.; Dent, E.; Alkhouri, H.; Kellett, J.; Williamson, M.; Asha, S.; Holdgate, A.; Mackenzie, J.; Winoto, L.; Fajardo-Pulido, D.; Ticehurst, M.; Hillman, K.; McCarthy, S.; Elcombe, E.; Rogers, K.; Cardona, M. | 2019 | Which frailty scale for patients admitted via Emergency Department? A cohort study | Prospective nested cohort study | To assess the ability of frailty to predict post-discharge adverse events, and evaluate the Clinical Frailty Scale (CFS) as e as a decision-making tool compared to clinical judgement. | N=4 (teaching hospitals) | Adverse events after admission | Frailty screening can help predict poorer outcomes, post-discharge and help discharge decisions. However the CFS was not optimal for implementation in busy settings such as the ED. | Nurses (no N) | M=80, SD=8.3 (N=899) |
| Meagher, A. D.; Lin, A.; M; ell, S. P.; Bulger, E.; Newgard, C. | 2019 | A Comparison of Scoring Systems for Predicting Short- and Long-term Survival After Trauma in Older Adults | Retrospective cohort study | Compare the prognostic value of five risk score tools, in predicting 30-day mortality risk amongst geriatric patients, as well as developing a decision rule | N=49 | Patient outcomes (30-day mortality) | The developed risk-prediction tool, overall performed better than the five existing ones, but emphasize that there is still a need for an ideal prognostic tool. | Emergency Medical Services (EMS) | M=81.7 (N=4849) |
| Seuren, Lucas M.; Stommel, Wyke; van Asselt, Dieneke; Sir, Özcan; Stommel, Martijn; Schoon, Yvonne | 2019 | Multidisciplinary meetings at the emergency department: A conversation-analytic study of decision-making | Conversation analysis of recorded multidisciplinary meetings (N=20) | To describe the organization and interactions involved in shared decision-making meeetings in multidisciplinary teams | N=1 | Not applicable | A structure arose naturally and was consistent throughout all meetings. Particularly, geriatric issues were helped to brought into the light by ad-hoc meetings, where especially frailty was the closing argument when choosing between surgical or conservative treatment | Physicians (N=3, EM, geriatrician and surgeon) | M=82.3, SD=6.2 (N=20) |
| Popovich, I.; Szecket, N.; Nahill, A | 2019 | Framing of clinical information affects physicians' diagnostic accuracy | Case-based decision-making analysis of diagnostic decisions | To explore framing bias in regards to differential diagnosis and its relation to diagnostic error | ED cases | Record of physician impression and their diagnostic plan | Differential diagnostic processes were affected by framing bias, causing diagnostic errors | Physicians (N=69) | 2/3 cases were geriatric (65 and 86 years) |
