# Supplementary material for: Conceptualizations of clinical decision-making: a scoping review in geriatric emergency medicine
Source: BMC Emerg Med. 2020 Sep 14;20:73. doi: 10.1186/s12873-020-00367-2 (PMC7489001; doi:10.1186/s12873-020-00367-2)
Supplement: Supplementary file 2 — Additional file 2. Coding of operationalizations of Clinical Decision-Making in geriatric emergency medicine. [file 12873_2020_367_MOESM2_ESM.doc]

***Appendix 2: Coding of operationalizations of Clinical Decision-Making in geriatric emergency medicine***

| *Overarching theme* | *Subthemes* | *ID* | *Operationalization of theme* |
| --- | --- | --- | --- |
| *CDM as the dispositional decisions* | Binary CDM | 42 | "…followed by a question asking if the physician completing the questionnaire would cease or continue CPR under that set of circumstances." (P12) |
|  |  | 16 | "The admission decision is regressed on the control, information availability, and use variables with SPSS 20. As the admission decision is represented by binary variable, we use mixed-effects logistic regressions...” (P1032) |
|  |  | 19 | "The decision to order physical restraint…" (P1280) |
|  | Categorical CDM | 21 | They “…asked EPs for the specific clinical information that most influenced their decision to order (or not to order) a CT.” (P291). |
|  |  | 6 | Decision-making refer to which specific decision was made based on the clinical data available: "…there also were instances when the clinician decision making was contrary to the absence of an AMI." (P1226) |
|  |  | 28 | They provided clinicians with a 15-item semi-structured interview tool comprised of open-ended questions investigating the role of self-efficacy and reflectivity, and a Likert-scale measures of confidence in regards to specific decisions made. |
|  |  | 41 | A description of when and how decisions are made. |
|  |  | 8 | Likert scale responses to everyday practice around history taking and physical exams. No mention of decision-making other than diagnostic errors, which is self-reported. |
|  |  | 45 | Describes decision-making in terms of styles of decision-making, measured by observations. |
|  |  | 51 | Record of treatment decisions and an investigation of practices amongst Multidisciplinary Team Meetings, when making these treatment decisions. |
|  |  | 52 | Record of diagnostic decision made, and the impact of framing bias. |
| *CDM as cognitive processes* | Cognitive processes e.g.: Illness scripts (networks of knowledge), Mental models, memory, judgement, human judgement/heuristic judgement/mental shortcuts. | 13 | "In making [treatment decisions, physicians] consider the disease, patient circumstances, and patient perceptions, as well as other factors. […] physicians engage in a large amount of mental processing [and] are often constrained by bounded rationality and satisficing..." (P154-155) |
|  |  | 26 | Describes decision making as 'mental models' which is further described as thought processes. It refers to former studies describing "…how norms might affect hospital-based physician's decision-making heuristics, case perceptions, and the consequential diagnosis and treatment..." (P345) |
|  |  | 29 | ""Clinical experience" consists of several components: [e.g.] accumulated knowledge [and] skill in collecting historical data... Knowledge is accumulated more or less [as a] data bank. Biases of availability, representativeness, and anchoring have been shown to be relevant, but it is not clear how much they detract from the value of "experience"." (P163) |
|  |  | 33 | "…nurses' judgements are not based solely on a static phenomenon of pre-existing patient criteria, but come to be revised as the performance is played out throughout the interaction." (P2449) |
|  |  | 35 | Builds upon several theories but concludes by formulating a model, which "...recognizes the salience of individual cognition, as well as acknowledging that the knowledge and experience that guides that cognition is constructed through social interaction and organizational context." (P161) |
|  |  | 30 | "…the ways in which the cognitive processes were used to solve the clinical problem had an enormous impact on the diagnostic error. The overreliance on the use of patterns was crucial." (P1280) |
|  |  | 32 | "Cognitive faculties deserve particular attention, as they are the bases of the clinical decision-making process… human abilities are limited and both gathering and retrieving information are inaccurate processes [2, 9]. Furthermore, in emergency medicine, "a priori" probabilities often are unknown, whereas missing data and ambiguities are frequent... This particular field favors intuitive and automatic tools as heuristics [1, 5]." (P2031) |
|  |  | 17 | "Heuristics are mental shortcuts that often produce valid judgements but can lead to errors in atypical or rare events. Because they reflect natural processes, heuristics are not easily, or even productively, replaced." (P9204) |
|  |  | 34 | "Clinicians also use heuristic observation of objective factors and application of scientific data, but also 'tacit' knowledge based on acquired expertise and pattern recognition." (P116) |
|  | Knowledge and attitudes | 3 | "We designed a comprehensive written survey to assess ED provider knowledge, attitudes, and practice regarding placement of IUCs [including] team dynamics of decision making in UIC placement and management…" (P415) |
|  |  | 15 | Refers to confidence, attitudes and knowledge, but does not address decision-making, specifically. |
|  |  | 46 | "The interview guide explored participants’ knowledge and behaviours towards URTI treatment, perceived norms of managing URTIs, and views on antibiotic resistance..." (P2) |
|  | Uncertainty | 25 | Diagnostic uncertainty: "…was quantified by a visual analogue scale (VAS) for ACS probability ranging from 0 to 100%." (P29 |
| *CDM as a model* | Statistical model/clinical decision rule | 14 | A decision-making analysis of certain risk stratification scores, as a statistical model. |
|  |  | 22 | Clinical decision rule consisting of blood culture data, i.e. basic guidelines. |
|  |  | 7 | A validation study of an assessment model. |
|  |  | 50 | Decision tree analysis of patient outcomes in order to develop a prediction tool/decision aid. |
|  | Decision rule and motivations/perception of utility | 39 | Validation of a decision rule and investigation of the motivations for certain decisions. |
|  |  | 44 | Describes decision-making in terms of a decision-making support tool and its utility. |
|  |  | 48 | Describes clinician’s views on a decision support tool for end-of-life treatment decisions. |
| *CDM as clinical judgement* | Clinical judgement: use of a structure/tool | 43 | "Upon final ED disposition, study staff administered a survey to the attending ED physician or senior resident querying the physician's impression of the likelihood of an acute bacterial infection and the infections suspected on a 5-point Likert scale from very unlikely to very likely." (P1803) |
|  |  | 11 | Clinical judgement: measured by the "CSHA-CFS" which classifies patients' level of frailty based on clinician’s clinical judgement. |
|  | Clinical Judgement: Practice as usual | 38 | Clinical decision-making tool and clinical judgement (practice as usual) assessed through chart data, but no specific information is given of how they assess the latter. |
|  |  | 1 | Operationalize clinical judgement as ED physicians’ probability estimate of heart failure (0-100%), clinical diagnosis by the ED physician, and a final diagnosis by a independent interdisciplinary panel. |
|  |  | 5 | "…identification of sepsis is based on clinical judgement, in turn, based on experience and diagnostic criteria according to guidelines…" (P2) |
|  |  | 18 | The clinician’s judgement of likelihood of heart failure being the cause of dyspnoea in the patient. |
|  |  | 36 | Comparing two measures' impact on "practice as usual": Anamnestic and diagnostic information was recorded, as well as the ED physician's initial clinical assessment (AKI or NO AKI), and level of confidence (0-100%). |
|  |  | 37 | "Because, to the best of our knowledge, no validated scoring system exists to quantify clinical judgement, we a priori chose to use the disposition decision of the treating physician in the ED as a proxy measure for clinical judgement…" (P294) |
|  |  | 2 | "clinical gestalt" is only mentioned as clinical judgement, but no further explanation |
|  |  | 4 | "Practice as usual", measured as only the specific risk assessment made by the attending physician. |
|  |  | 9 | Prediction tool compared to clinical judgement: judgement of likelihood of a certain diagnosis |
|  |  | 20 | Uses decision statistics, and Clinical Judgement as practice as usual, measured as the probability of a disease on a visual analogue scale |
|  |  | 23 | Clinical judgement determined by likelihood of a specific diagnosis and evaluating clinical assessment and diagnosis. |
|  |  | 27 | Clinical uncertainty (measured as a likelihood score) and outcome measures such as length of hospital stay, etc. together comprises clinical judgement. |
|  |  | 10 | Comparing a risk stratification tool (MEWS) to clinical judgement, defined as: "…the normal practice by nurses using individual's nursing knowledge, clinical expertise and gut feeling - judging based on strong feelings rather than facts, plus the measurement of 3 vital signs - blood pressure, pulse and body temperature, for clinical decision-making." (10:P27) |
|  |  | 12 | "practise as usual" based on chart reviews. |
|  |  | 31 | clinical judgement was just unaided judgement, presumably relying on experience and knowledge but this is not specifically addressed |
|  |  | 40 | They describe 'unstandardized clinical judgement' which seems to refer to the 'non-stress-tested' group. As such, it seems only to refer to the observation group - i.e. "practice as usual" |
|  |  | 24 | "Clinical judgement can be defines as "an interpretation or conclusion about a patient's needs, concerns, or health problems, and/or the decision to take action (or not), use or modify standard approaches, or improvise new ones as deemed appropriate by the patient's response" [11]. It is complex and requires a flexible ability to recognise prominent aspects of an undefined clinical situation interpret their meaning and respond appropriately. It relates to the experience of individual clinicians." (P5) |
|  |  | 47 | Prediction tool compared to clinical judgement, reported by patient outcome. |
|  |  | 49 | One of the measures relied on clinician's clinical judgement, i.e. "eye-balling" the state of frailty |
